# A Bayesian Framework for Single Image Dehazing considering Noise

**DOI:** 10.1155/2014/651986

**Published:** 2014-08-19

**Authors:** Dong Nan, Du-yan Bi, Chang Liu, Shi-ping Ma, Lin-yuan He

**Affiliations:** Institute of Aeronautics and Astronautics Engineering, Air Force Engineering University, No. 1 Baling Road, Baqiao District, Xi'an 710038, China

## Abstract

The single image dehazing algorithms in existence can only satisfy the demand for dehazing efficiency, not for denoising. In order to solve the problem, a Bayesian framework for single image dehazing considering noise is proposed. Firstly, the Bayesian framework is transformed to meet the dehazing algorithm. Then, the probability density function of the improved atmospheric scattering model is estimated by using the statistical prior and objective assumption of degraded image. Finally, the reflectance image is achieved by an iterative approach with feedback to reach the balance between dehazing and denoising. Experimental results demonstrate that the proposed method can remove haze and noise simultaneously and effectively.

## 1. Introduction

As one of the most important topics and basic issues in image processing, single image dehazing aims at two aspects. One is creating visually pleasing images suitable for human visual perception; the other is improving the interpretability of images for computer vision and preprocessing tasks. Thus, advanced techniques of single image dehazing are in urgent needs. The existing papers can be roughly classified into two methods. The first scheme based on image enhancement technique aims at improving the visual effect of image directly, such as gamma correction [1], histogram equalization [2], and Retinex [3, 4]. This scheme is fast and simple but has strong pertinence and can hardly adjust all image characteristics to a proper range simultaneously, according to human vision system. The second one is based on image restoration technique. Strong prior or assumption atmospheric transmission and environmental luminance model makes it possible to solve the problem caused by the atmospheric scattering which has the ill-posedness, for instance, Tan [5] optimization based on Markov random field (MRF), Fattal [6] estimation based on independent component analysis (ICA), and He et al. [7] solution based on dark channel prior (DCP). This scheme is a hotspot recently, but it is overly dependent on the model and vulnerable by external environment [8–11].

By analyzing the recent dehazing algorithms based on image restoration, we find that most algorithms only consider improving contrast and luminance of degraded image; however, in fact, noise is a universal phenomenon and a significant issue in dehazing [12–17]. In 2012, Fang et al. [15] realized simultaneous dehazing and denoising based on joint bilateral filter [16], but it may cause excessive enhancement as the parameters of joint bilateral filter are unknown. In the same year, Matlin and Milanfar [17] proposed two methods for removing haze and noise from a single image: one is to denoise the image prior to dehazing based on BM3D [18] and He's algorithm; the other is an iterative regression method. Both of them perform well when the noise level is precisely known, but when the noise level is not given, latent errors from either “under”-denoising or “over”-denoising can be amplified. In 2013, Lan et al. [19] presented a haze image model considering both sensor blur and noise. Based on the degradation model, a three-stage haze removal algorithm is proposed; the algorithm is effective, but it denoises the image prior to dehazing, which would cause a loss of information on image details.

In this paper, we propose a novel “Bayesian framework,” which would avoid dynamic range compression in He's algorithm. The accuracy of the input image is ensured by removing haze and noise simultaneously. The robustness of our approach is guaranteed by the iterative approach with feedback. This paper is arranged as follows.  Section 2 describes the development of image dehazing and proposes an improved atmospheric scattering model based on McCartney's method. In Section 3, a Bayesian framework for single image dehazing considering noise is proposed. The experiments are presented in Section 4. Conclusion is summarized in Section 5.

## 2. Backgrounds

The single image dehazing algorithm was classified as an image enhancement technique in the earlier time. Middleton [20] modeled it as an image restoration technique in 1952, and then McCartney [21] developed it to a mature model based on Rayleigh scattering, which was widely used to describe the formation of the degraded image in 1976. In this section, we will make a brief introduction of the McCartney's atmospheric scattering model and propose our improved atmospheric scattering model based on its defects.

### 2.1. The McCartney's Atmospheric Scattering Model

As is well known, the image received by a sensor from scene points is often absorbed and scattered by a complex medium. In computer vision and atmospheric optics, the McCartney's atmospheric scattering is playing a major role in image degradation. It was modeled as follows [22]:

(1) $$\begin{array}{c} I\left(x,y\right)=t\left(x,y\right)J\left(x,y\right)+\left(1-t\left(x,y\right)\right)A, \end{array}$$

where *I*(*x*, *y*) denotes the observed degraded image. *J*(*x*, *y*) denotes the scene radiance, which represents original appearance of image. *A*, the global atmospheric light, is mostly recognized as the mean of the top 0.6% brightest pixels in the haze image [15]. *t*(*x*, *y*) is the atmospheric transmission map. Then, the problem changes into how to estimate the latent image *J*(*x*, *y*) from *I*(*x*, *y*) when *t*(*x*, *y*) are given, which makes it an abnormal equation.

### 2.2. The Improved Atmospheric Scattering Model

Noise from environment and sensor is also an important degradation factor, but it is not considered in McCartney's atmospheric scattering model. Therefore, our improved atmospheric scattering model is proposed as follows:

(2) $$\begin{array}{c} I\left(x,y\right)=t\left(x,y\right)J\left(x,y\right)+\left(1-t\left(x,y\right)\right)A+n\left(x,y\right), \end{array}$$

where *n*(*x*, *y*) denotes zero-mean Gaussian noise, as it comes from environment and sensor [25, 26]. There are two kinds of approaches to solve (2): one is to dehaze and denoise step by step; the other is to dehaze and denoise simultaneously. The former includes denoising prior for dehazing and dehazing prior for denoising. Denoising prior for dehazing may cause loss of information on image details. Dehazing prior to denoising can be explained as follows:

(3) $$\begin{array}{c} J\left(x,y\right)=A+\frac{I\left(x,y\right)-A}{t\left(x,y\right)}-\frac{n\left(x,y\right)}{t\left(x,y\right)}, \end{array}$$

where *t*(*x*, *y*) is a value between 0 and 1, and it varies inversely with the density of haze. Equation (3) implies that the noise will be amplified if not removed before dehazing, especially in very hazy regions where *t*(*x*, *y*) is close to 0 and the noise contribution can dominate the results. Therefore, the main focus of our work is to dehaze and denoise simultaneously.

## 3. Our Approach

The key to our approach is that it combines the best of the Bayesian framework, the statistical prior and objective assumption of degraded image, and the iterative algorithm with feedback, to achieve the balance between dehazing and denoising. This section arranges as follows. The establishment of dehazing based on Bayesian framework is in Section 3.1, the definition of Bayesian framework's probability density function in Section 3.2, and the solution of our approach in Section 3.3.

### 3.1. Image Dehazing Based on Bayesian Framework

Rearranging (2), we find the following expression:

(4) $$\begin{array}{c} I\left(x,y\right)-A=\left(J\left(x,y\right)-A\right)t\left(x,y\right)+n\left(x,y\right). \end{array}$$

In order to keep (4) nonnegative, we reverse it as

(5) $$\begin{array}{c} I_{A}\left(x,y\right)=J_{A}\left(x,y\right)t\left(x,y\right)-n\left(x,y\right), \end{array}$$

where *I*_*A*_(*x*, *y*) = *A* − *I*(*x*, *y*), *J*_*A*_(*x*, *y*) = *A* − *J*(*x*, *y*). According to Bayesian law, posterior probability is defined [27] as

(6) $$\begin{array}{c} p\left(J_{A},t\;|\;I_{A}\right)=\frac{p\left(I_{A}\;|\;J_{A},t\right)p\left(J_{A}\;|\;t\right)p\left(t\right)}{p\left(I_{A}\right)}, \end{array}$$

where *p*(*I*_*A*_) is a constant, as *I*_*A*_ is given; *p*(*J*_*A*_∣*t*) = *p*(*J*_*A*_), as *J*_*A*_ and *t* are uncorrelated. In order to get *J*_*A*_ and *t*, we can maximize (6) as follows:

(7) (J^A,t^)=argmax⁡(JA,t)[p(JA,t ∣ IA)]=argmax⁡(JA,t)[p(IAJA,t)p(JA)p(t)].

### 3.2. The Obtaining of Probability Density Function

#### 3.2.1. The Obtaining of the Prior Probability Density Function Based on Noise Level Estimation

Assuming that the signal and the noise are uncorrelated, the variance of (5) on direction *u* can be expressed as

(8) $$\begin{array}{c} V\left(u^{T}I_{A}\right)=\text{V}\left(u^{T}J_{A}t\right)+\sigma^{2}, \end{array}$$

where *V*(*x*) represents the variance of the dataset *x*; *σ* is the standard deviation of the Gaussian noise. We define the minimum variance direction *u*_min⁡_ as

(9) $$\begin{array}{c} u_{\min}=\arg\;\underset{u}{\min}\;V\left(u^{T}I_{A}\right)=\arg\;\underset{u}{\min}\;V\left(u^{T}J_{A}t\right). \end{array}$$

The variance of *I* can be calculated using principal component analysis (PCA) [28]

(10) $$\begin{array}{c} V\left(u_{i}^{T}I\right)=u_{i}^{T}\Sigma_{I}u_{i}=\overset{P}{\underset{j=1}{\sum }}u_{i}^{T}\lambda_{j}u_{j}u_{j}^{T}u_{i}=\lambda_{i}{\left(u_{i}^{T}u_{i}\right)}^{2}=\lambda_{i}, \end{array}$$

where *u*_*i*_^*T*^ *u*_*i*_ = 0, when *i* ≠ *j*; Σ_*I*_ denotes the covariance matrix of *I*; *λ*_*i*_ represents the *i*th eigenvalue of the matrix Σ_*I*_. The variance of the data projected onto the minimum variance direction equals the minimum eigenvalue of the covariance matrix. Then we can derive (8) as follows:

(11) $$\begin{array}{c} \lambda_{\min}\left(\Sigma_{I_{A}}\right)=\lambda_{\min}\left(\Sigma_{J_{A}t}\right)+\sigma_{n}^{2}\left(I\right). \end{array}$$

The noise level can be estimated easily if we can decompose the minimum eigenvalue of the covariance matrix of the noisy patches as (11). The weak textured patches are known to span only low dimensional subspace. The minimum eigenvalue of the covariance matrix of such weak textured patches is approximately zero. Then, the noise level can be estimated simply as follows:

(12) $$\begin{array}{c} {\hat{\sigma }}_{n}^{2}\left(I\right)=\lambda_{\min}\left(\Sigma_{I^{\prime }}\right), \end{array}$$

where Σ_*I*′_ is the covariance matrix of the selected weak textured patches. We can select the weak textured patches as [29]. After acknowledging the noise level, we model the inherent noise in the observations with Gaussian distribution of the same variance *σ* ^2^. The likelihood of *p*(*I*_*A*_∣*J*_*A*_, *t*) then becomes

(13) p(IA ∣ JA,t)=12πσ2exp⁡[−||IA(x,y)−JA(x,y)t(x,y)||222σ2],

where the prior probability density function in RGB channels are independent of the randomness of noise distribution.

#### 3.2.2. The Obtaining of* J*_*A*_'s Probability Density Function Based on the Distribution of Chromaticity Gradient Histogram

**Figure 1:**
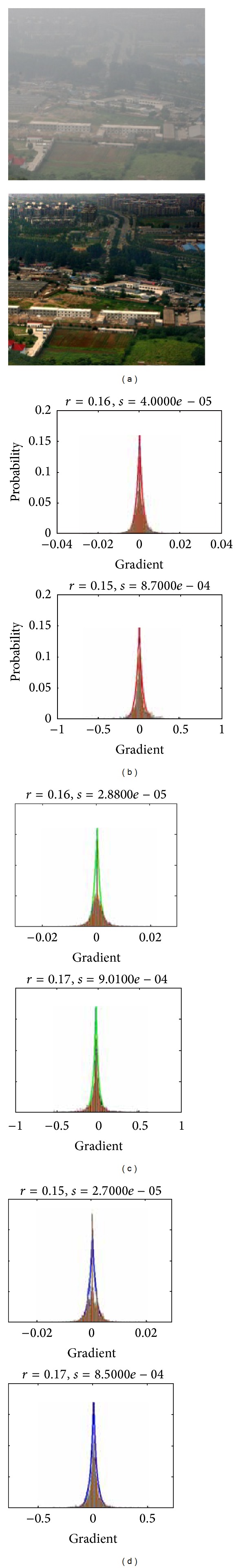
Distribution of chromaticity gradient histogram. Top: the haze image. Bottom: the haze-free image (the horizontal gradient is shown in the figure; the vertical gradient has the same character as it). (a) The haze image and its haze-free image [7], (b) the distribution of chromaticity gradient histogram of red light component, (c) the distribution of chromaticity gradient histogram of green light component, and (d) the distribution of chromaticity gradient histogram of blue light component.

**Figure 2:**
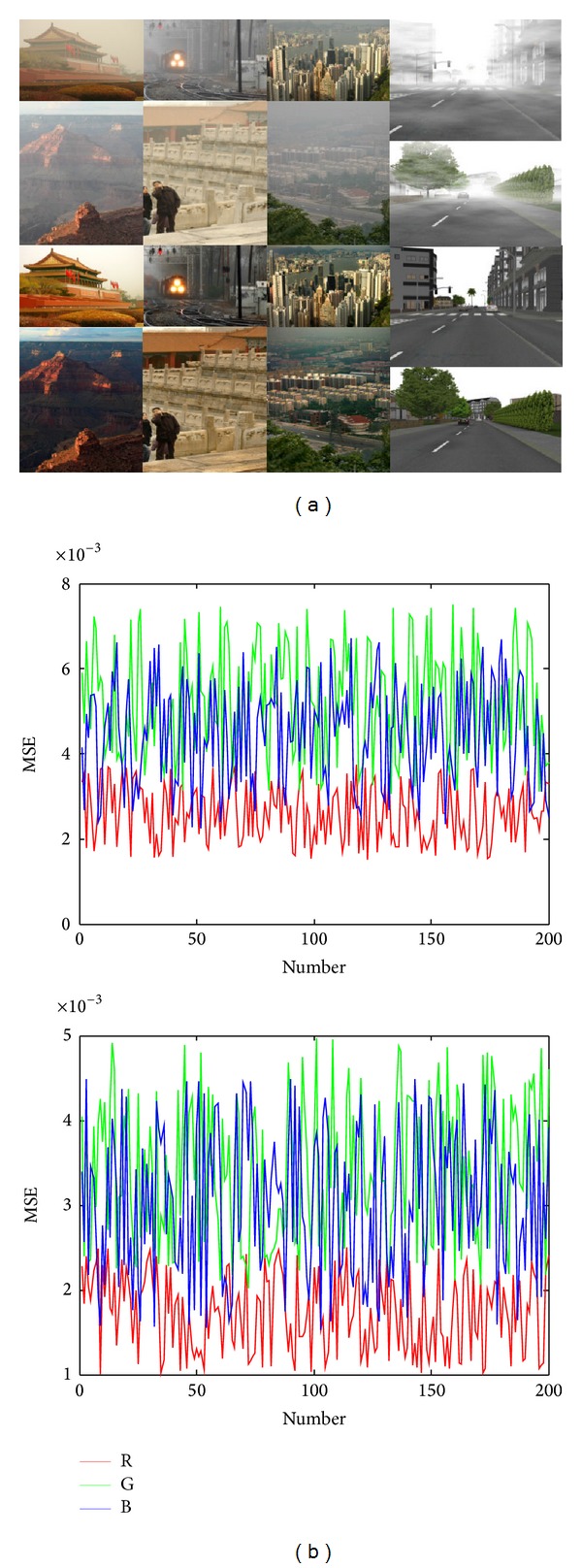
Results of MSE. Top: the haze image. Bottom: the haze-free image. (a) Example for images in our haze and haze-free image database and (b) the MSE between the distribution of chromaticity gradient histogram and their exponential power distribution of the 200 haze images and their haze-free images.

After analyzing 200 randomly selected haze images and their haze-free images, we can find that the distribution of chromaticity gradient histogram of haze images is the same as their haze-free images, which is the power of the exponential power distribution. In order to explain this, we can define the chromaticity of input image *I*(*x*, *y*) as follows [27]:

(14) $$\begin{array}{c} C^{I^{k}}\left(x,y\right)=\frac{I^{k}\left(x,y\right)}{\sum_{k\in \left\{R,G,B\right\}}^{}I^{n}\left(x,y\right)}, \end{array}$$

where *k* ∈ {*R*, *G*, *B*}. The gradient of *I*(*x*, *y*) is defined as

(15) $$\begin{array}{c} G_{I}\left(x,y\right)=\left[\begin{array}{cc} D_{h}I\left(x,y\right)\text{,} & D_{v}I\left(x,y\right) \end{array}\right], \end{array}$$

where *D*_*h*_ and *D*_*v*_, respectively, represent the matrices of horizontal and vertical derivative operators. For example, the distributions of chromaticity gradient histogram of haze images and their haze-free images are shown in Figure 1. We can find that all of them are exponentially distributed; the only difference is that they have different rate parameter *r* and normalization parameter *s*.  Figure 2 shows mean squared error (MSE) between the distribution of chromaticity gradient histogram and their exponential power distribution of the 200 haze images and their haze-free images. The result demonstrates the reliability of fitting by the exponential power distribution, as their MSE are still in the low level.

Therefore,* J*_*A*_'s probability density function can be obtained as follows:

(16) $$\begin{array}{c} p_{k}\left(J_{A}\right)=p\left(G_{C^{J_{A}^{k}}}^{h}\right)=s\exp\left[-r\left|G_{C^{J_{A}^{k}}}^{h}\left(x,y\right)\right|\right], \end{array}$$

where *k* ∈ {*R*, *G*, *B*} and *r* and *s*, respectively, represent rate parameter and normalization parameter of the exponential power distribution.

#### 3.2.3. The Obtaining of *t*'s Probability Density Function Based on the Sensitivity of Green Wavelength

**Figure 3:**
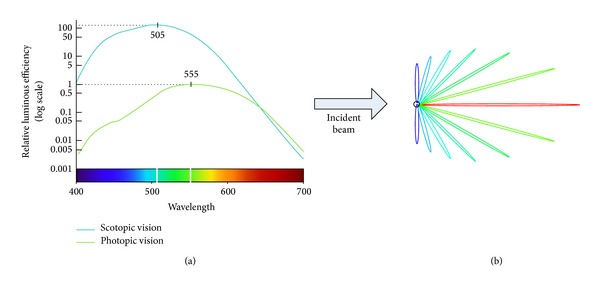
The sensitivity of green wavelength. (a) Photonic and scotopic response of the HVS [23] and (b) angular patterns of forward-scattered intensity from particle of aerosol [24].

Human visual system (HVS) has specific response sensitivity to the small interval of light wavelength [30].  Figure 3(a) shows the segment of wavelength where the HVS has its maximum sensitivity. In this figure one curve represents the sensitivity for photonic vision and the other for scotopic vision. Because of the much higher sensitivity to luminous efficiency of the scotopic vision compared to the photopic vision, both of them have maximum sensitivity from green-blue wavelength for red and blue perception, and the combined overall sensitivity ranges from 505 nm to 555 nm.  Figure 3(b) shows the symmetric forward-scattered intensity from particle of aerosol in the incident light beam: the blue wavelength will tendentially be scattered more into 90° (270°, resp.) direction relative to the incident light in the plane of observation; the red wavelength will be scattered into forward (0°) in the plane of observation. With the angular increases from 0° to 90°, the intensity will be decreased. Meanwhile, the light wavelength ranges from red to blue. Due to the response of green wavelength and the intensity of forward-scattered, the green light component of image is assumed as the input of *t*, which can not only satisfy efficiency (reduce the numbers of the estimation of transmission map from three to one) but also corresponds to the statistical prior.

In order to meet the global spatial smoothness of the image, which is the basic assumption of the atmospheric transmission map, meanwhile, to preserve the detail-and-edge information of *J*_*A*_ when denoising, we combine characters of both the sensitivity of green wavelength and the bilateral filtering to estimate the initial atmospheric transmission map *t*, which can be formulated as follows [31]:

(17) tG(x,y)=1W∑(xp,yp)∈sGσs(||(x,y)−(xp,yp)||)×Gσr(||IG(x,y)−IG(xp,yp)||)IG(xp,yp),W=∑(xp,yp)∈SGσs(||(x,y)−(xp,yp)||)    ×Gσr(||IG(x,y)−IG(xp,yp)||),

where *I* ^*G*^(*x*, *y*) is the green light component of haze image, *W* is normalization parameter, *S* is a local patch centered at (*x*, *y*) with 7 × 7, and *G*_*σ*_*s*__(•) and *G*_*σ*_*r*__(•), respectively, represent spatial and luminance function; they can be defined [32] as:

(18) Gσs(||(x,y)−(xp,yp)||) =exp⁡(−|x−xp|2+|y−yp|22σs2),Gσr(||IG(x,y)−IG(xp,yp)||) =exp⁡(−|IG(x,y)−IG(xp,yp)|22σr2),

where *σ*_*s*_ and *σ*_*r*_, respectively, represent the standard deviation of spatial and luminance function. According to the exponential damping of *t* [33], *t*'s probability density function is formulated as follows:

(19) $$\begin{array}{c} p\left(t\right)=\frac{1}{\sqrt{2\pi \sigma_{t_{G}}^{2}}}\exp\left[-\frac{{||t\left(x,y\right)-t_{G}\left(x,y\right)||}_{2}^{2}}{2\sigma_{t_{G}}^{2}}\right], \end{array}$$

where *σ*_*t*_*G*__ is the standard deviation of the initial estimation of *t*_*G*_, which can be calculated by (12).

### 3.3. The Iterative Approach with Feedback Based on the Law of Minimum Noise Level

Putting the likelihood of (13), (16), and (19) into (7), we can estimate *J*_*A*_ and *t* simply by

(20) (J^A,t^)=argmin⁡(JA,t)[∑k∈{R,G,B}(||IAk(x,y)−JAk(x,y)t(x,y)||222σ2+λ|GCJAkh(x,y)|)+||t(x,y)−tG(x,y)||222σtG2].

Optimizing (20) directly is not possible, as *J*_*A*_ and *t* are unknown at the same time. In order to solve this, we can estimate each variable with the other one fixed. Thus, (20) becomes two separate partial energies minimized functions as follows:

(21) J^A=arg min⁡JA[∑k∈{R,G,B}(||IAk(x,y)−JAk(x,y)t(x,y)||222σ2+λ|GCJAkh(x,y)|)],

(22) t^=arg min⁡t[∑k∈{R,G,B}(||IAk(x,y)−JAk(x,y)t(x,y)||222σ2)+||t(x,y)−tG(x,y)||222σtG2].

**Figure 4:**
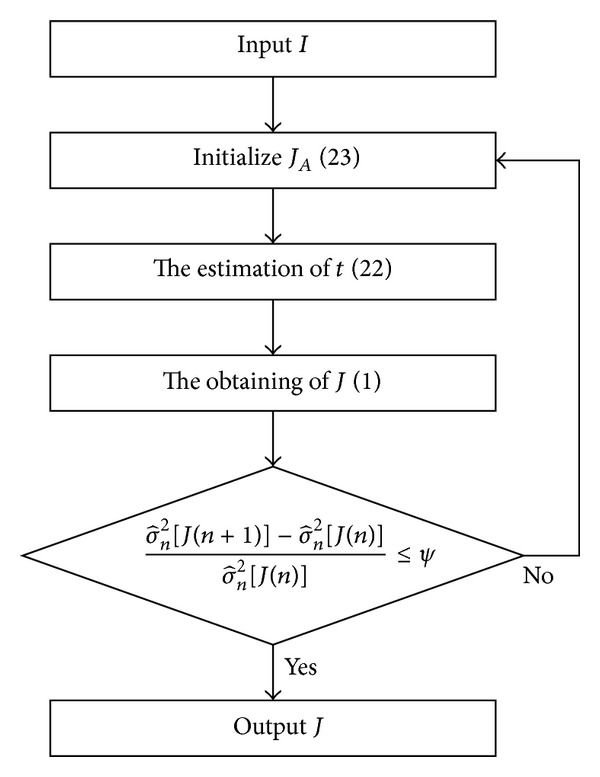
The iterative approach with feedback based on the law of minimum noise level, where *ψ* = 0.03 is the terminating threshold. Generally, we will get a good result when the number of iterations is 3 (e.g., Figure 7). In Figures 5 and 6, “He-BM3D” is the result of dehazing by He et al. [7] prior to denoising by BM3D [18], which has the same noise level as our result with 3 iterations.

When solving (21), a large computational complexity is expected as we have to traverse every pixel's level of light in RGB channels simultaneously. In order to avoid this problem, we choose (22) to solve, as the transmission map *t* is the same in the three channels. We assume that *t*'s value is traversed between −5% and +5% of its initial value, which will improve the efficiency greatly. Considering dehazing, we fix *J*_*A*_ by BM3D [18]:

(23) $$\begin{array}{c} J_{A}^{n+1}={\left(J_{A}^{n}\right)}_{\text{BM}3\text{D}}, \end{array}$$

where *n* = 0,1, 2,…. According to (22) and (23), we can find two items in it: the first item assures more edge information in the transmission map; the second item ensures that more noise will be removed as the value comes near to *t*_*G*_. Finally, we adopt the iterative approach with feedback in Figure 4 to achieve the balance between dehazing and denoising.

**Figure 5:**
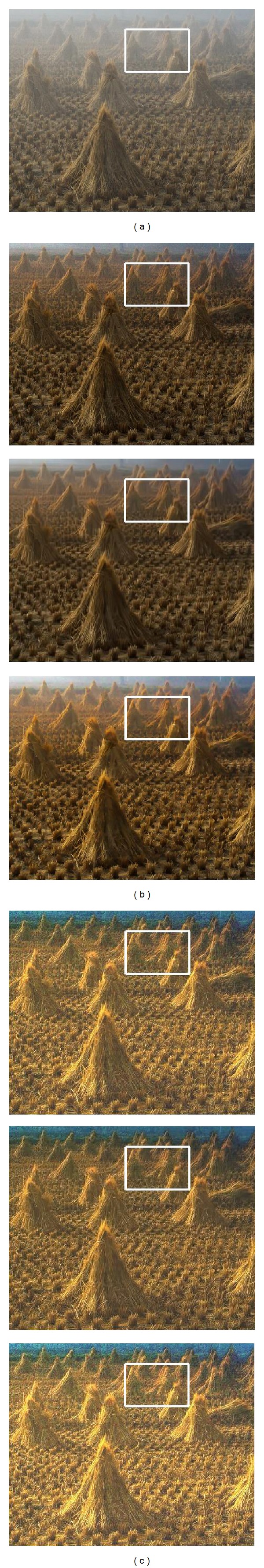
Natural images to test performance. (a) Input, (b) the contrast experiments (from top to bottom: He's result [7], He-BM3D's result, and Lan's result [19]), and (c) our result (from top to bottom: 1st iteration, 2nd iteration, and 3rd iteration).

**Figure 6:**
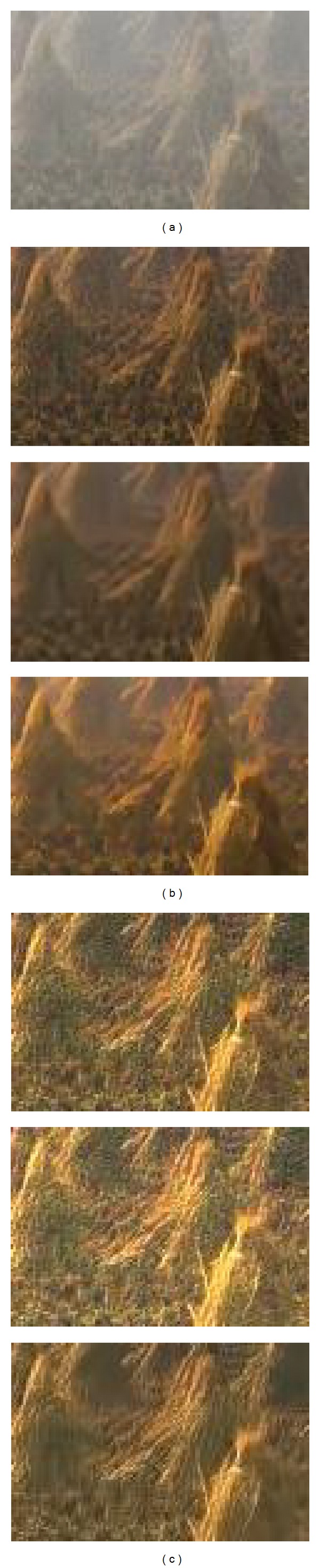
The enlargement of the area outlined in white of Figure 5. (a) Input, (b) the contrast experiments (from top to bottom: He's result [7], He-BM3D's result, and Lan's result [19]), and (c) our result (from top to bottom: 1st iteration, 2nd iteration, and 3rd iteration).

**Figure 7:**
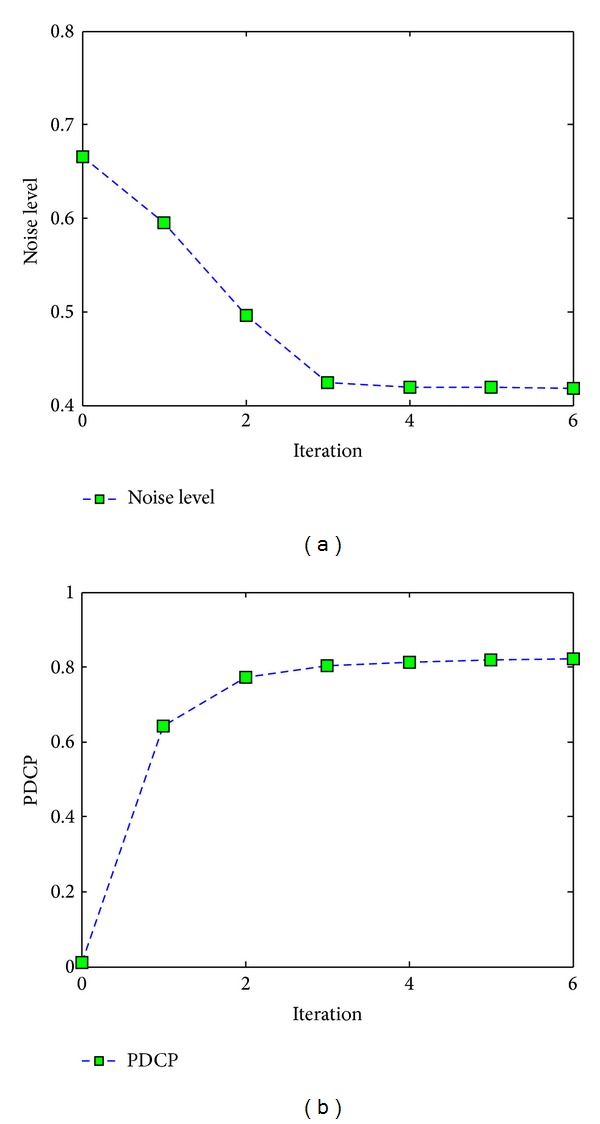
The relation curves. (a) The relation curve between numbers of iteration and noise level and (b) the relation curve between numbers of iteration and PDCP.

Figures 5 and 6 show that He's result can recover more details than our 3rd iteration's result, but with more noise; He-BM3D's result has the same dehazing effect as our 3rd iteration's result, but less edge and texture information; meanwhile, our result has a better dehazing effect than Lan's result with nearly the same noise level. Besides, our approach's relationship between iteration and the final restored effect of Figure 5(a) is shown in Figure 7. The “noise level” is estimated by (12), and a lower value suggests better effect of image denoising. The “PDCP” is the proportion of the number of pixels, whose luminance is lower than 25 in our DCP image; with the increase of the PDCP, we will have a better result of image dehazing. Finally, Figure 7 shows that when the number of iterations is 3, a stable and effective result will be achieved.

## 4. Experimental Results

**Figure 8:**
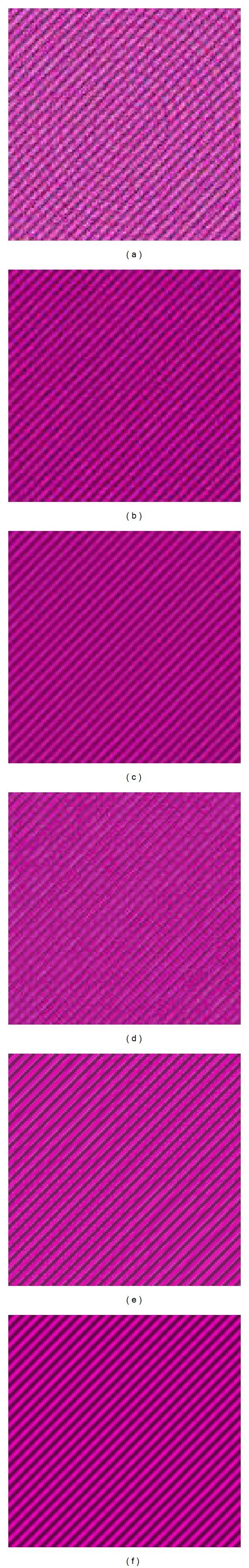
Synthetic images with haze and noise to test performance. (a) Input [6], (b) He's result [7], (c) He-BM3D's result, (d) Lan's result [19], (e) our result, and (f) the original image [6].

**Figure 9:**
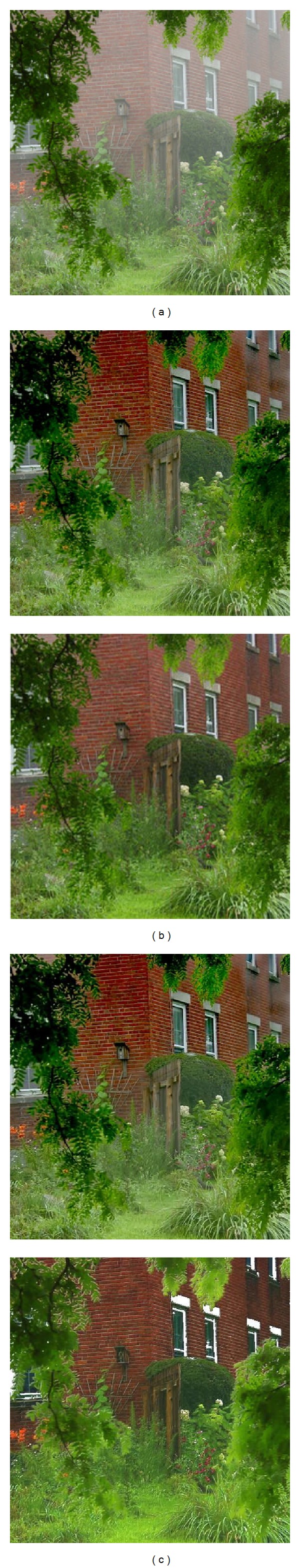
Close depth images to test performance. (a) Input, (b) top: He's result [7]; bottom: Lan's result [19], and (c) top: He-BM3D's result; bottom: our result.

**Figure 10:**
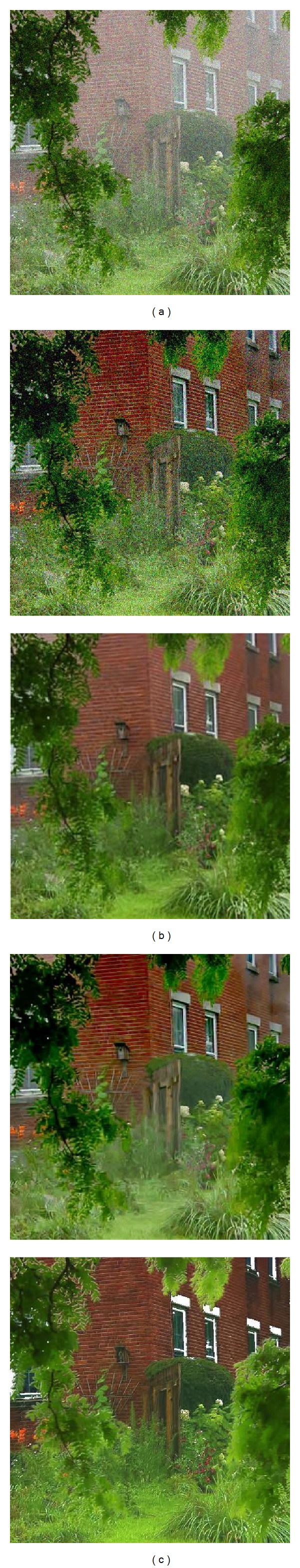
Close depth images with noise (0.2) to test performance. (a) Input, (b) top: He's result [7]; bottom: Lan's result [19], and (c) top: He-BM3D's result; bottom: our result.

**Figure 11:**
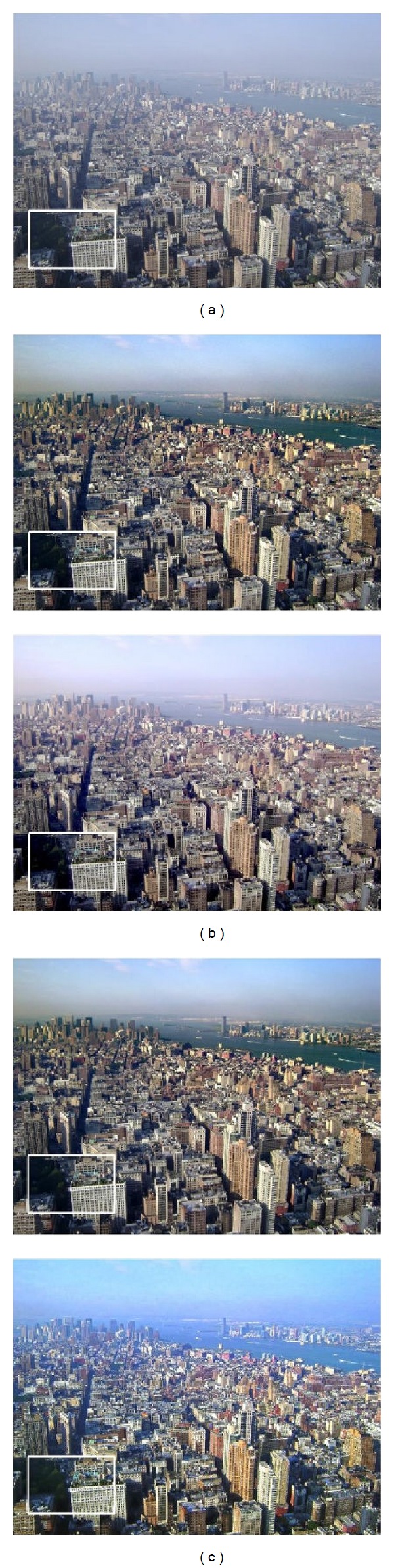
Deep depth images to test performance. (a) Input, (b) top: He's result [7]; bottom: Lan's result [19], and (c) top: He-BM3D's result; bottom: our result.

**Figure 12:**
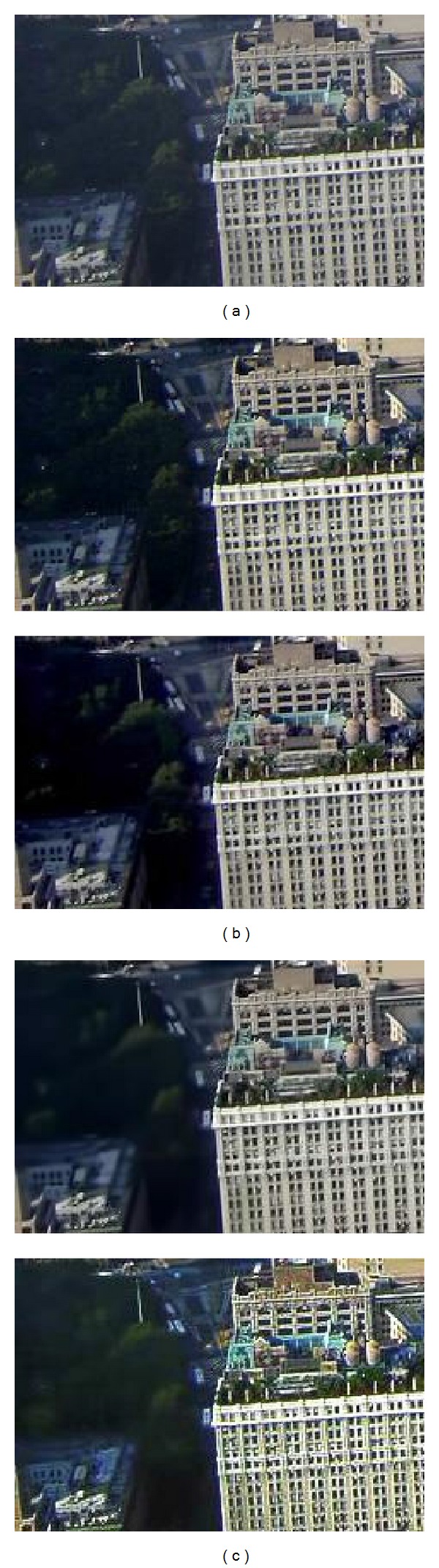
The enlargement of the area outlined in white of Figure 11. (a) Input, (b) top: He's result [7]; bottom: Lan's result [19], and (c) top: He-BM3D's result, bottom: our result.

In order to validate the performance of our approach, 4 groups of experiments are established: synthetic images with haze and noise to test performance in Figure 8, close depth images to test performance in Figure 9, close depth images with noise to test performance in Figure 10, and deep depth images and their local enlargements to test performance in Figures 11 and 12.

Figure 8 shows that our approach can remove haze and noise more effectively than others. Meanwhile, we apply PSNR as a typical objective evaluation listed in Table 1.

We compare the proposed algorithms with different condition in Figures 9 and 10. The results show that there are fewer details of the local dark areas in He's result, as He's algorithm could lead to lower mean luminance than original haze image; for example, the texture information of leaf in the close range is hidden after processing. In addition, when dealing with noise image, the noise contribution is amplified in He's result, which can dominate the result. He-BM3D's result has the same effect as He's result in Figure 9 but may cause more smoothing and detail loss after BM3D in Figure 10, as it aims to achieve the same noise level as our result. Lan's algorithm is to denoise prior to dehazing, which may cause a loss of information on details; the latent hazards may not be clear in Figure 9 but it is obvious in Figure 10. However, whether in Figure 9 or Figure 10, our approach achieves good effects both in dehazing and denoising, which demonstrates our approach's capacity in scene restoration and detail protection.

**Figure 13:**
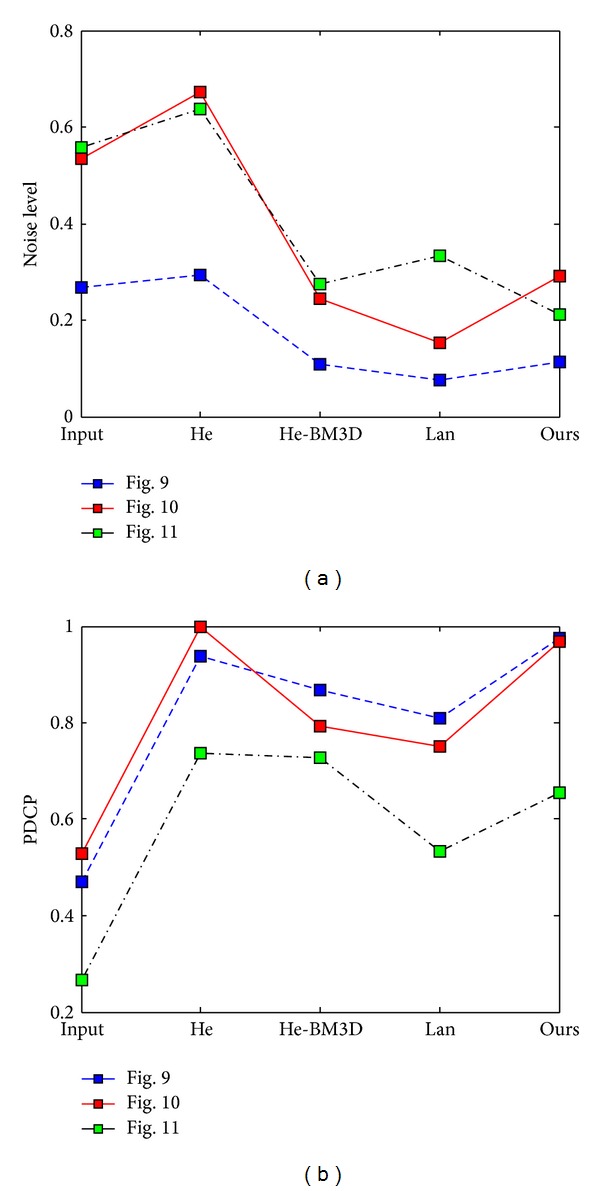
The objective evaluation. (a) Noise level and (b) PDCP.

Figures 11 and 12 show the deep depth image coming with lots of details and complex noise. He's algorithm may amplify the noise and lose texture information. Then, denoising by BM3D will lead to smoothing and detail loss. Even Lan's algorithm cannot restore scene and details in the large depth area effectively. Our result, in contrast, could present more vivid restored image with high contrast and obtain nearly the same haze-free image as He's result. In particular, the proposed algorithm achieves wider dynamic range compression in dark regions and also holds the strong ability of resisting noise. Except for subjective evaluation, the typical objective evaluation is shown in Figure 13. The figure shows that our result achieves almost the same noise level as Lan's result and gets nearly the same effect as He's result in dehazing. Thus, we can see that the subjective evaluation agrees with the objective one.

## 5. Conclusion

In this paper, we present a novel single image dehazing approach considering noise based on Bayesian framework. We focus on an improved atmospheric scattering model by considering noise and haze simultaneously. The likelihood of posterior probability based on Bayesian framework is estimated by the statistical prior and objective assumption of degraded image. Meanwhile, we focus more on the efficiency by choosing the transmission map to get the scene radiance. BM3D is used to fix the initial input of the iterative approach with feedback, which can help to achieve the balance between dehazing and denoising. The experimental results demonstrate that our approach is effective, especially in challenging scenes with both haze and noise. However, color distortion still exists which will be involved in our future work.

## Figures and Tables

**Table 1:** The PSNR of Figure 8.

Figure 8(a)	Figure 8(b)	Figure 8(c)	Figure 8(d)	Figure 8(e)

61.4638	64.1724	63.4186	61.2034	**67.7450**
